# From rhetoric to representation: athlete-centred governance understood, constructed, and enacted in a mega-sport event

**DOI:** 10.3389/fspor.2026.1864884

**Published:** 2026-07-01

**Authors:** Nicole W. Forrester

**Affiliations:** RTA School of Media, The Creative School, Toronto Metropolitan University, Toronto, ON, Canada

**Keywords:** athlete representation, athlete voice, athlete-centred, mega-sport event, process tracing, sport governance, stakeholder salience

## Abstract

While athlete-centredness is increasingly championed globally by sport organizations, the concept remains vaguely defined and inconsistently operationalized. This creates a systemic gap between rhetoric and the enactment of athlete voice in mega-sports events (MSEs) planning. Despite increasing emphasis on athlete-centred governance, limited empirical research has examined how athlete influence is operationalized within governance structures. This study examines how athlete-centred governance was understood, constructed, and enacted through Athlete Advisory Council (AAC) processes over time (2011–2015) in the Toronto 2015 Pan/Parapan American Games. Drawing on a qualitative case study design, the research utilizes interpretive process tracing and documentary analysis of organizational documents. The study employs the stakeholder identification and salience model to analyze the influence of athletes through power, legitimacy, and urgency. Reflexivity was employed to leverage the lead researcher's positionality as the former AAC vice-chair while maintaining analytical rigor. The findings reveal a five-phase lifecycle of athlete-centred governance. Results demonstrate that athlete-centred governance operated through embedded epistemic influence and structured operational integration rather than formal redistribution of decision-making power. Athlete legitimacy and urgency expanded through sustained horizontal and vertical integration while institutional authority remained centralized. Rather than functioning solely as symbolic representation, formal inclusion enabled athletes to contribute meaningfully to feasibility discussions, organizational planning, and operational delivery despite constrained formal authority. These findings position athlete-centred governance as a form of bounded empowerment in which influence is exercised relationally, operationally, and epistemically rather than primarily through formal voting power.

## 1 Introduction

Athlete-centredness has become an integral component of contemporary sport governance. Various sport governance initiatives associated with mega-sport events (MSEs), including the International Olympic Committee's (IOC) Olympic Agenda 2020 + 5 and Commonwealth Sport, have referenced it as a guiding governance principle (1–3). Despite its relevance, the concept of athlete-centredness remains vaguely defined and inconsistently operationalized (4–6). First popularized in coaching literature, athlete-centredness is often understood through a service delivery lens shaped primarily by those providing services to athletes (7, 8). However, athletes themselves are often excluded from defining what the concept means to them. AthletesCAN, the first athlete developed organization representing the views of national team athletes, has defined athlete-centredness as:

…both a concept and a process, rather than a single action or event. In an athlete-centred sport system, the values, programs, policies, resource allocation and priorities of sport organizations and agencies place primary emphasis on consideration of athletes’ needs in a holistic sense and performance goals within that context. Those responsible for leadership and decision-making in sport must include the athlete in both defining the needs and goals and in determining how to meet them; i.e., the athlete should be the active subject in, not the object of sporting programs (9).

Taken together, this framing highlights the need to critically examine how athlete-centredness is realized and implemented (4).

This paper examines how an MSE operationalizes athlete-centredness. Specifically, the implementation of athlete voice is examined using stakeholder theory and the stakeholder identification and salience framework. Stakeholder theory suggests that the success of an organization is shaped by its ability to create value by addressing stakeholder needs (10, 11). It emphasizes the importance of prioritizing and engaging stakeholders to balance competing needs effectively. The stakeholder identification and salience framework builds on this by examining how stakeholder influence is shaped through competing organizational priorities. This dominant framework conceptualizes salience as a function of power, legitimacy, and urgency (12). These attributes often intersect to shape stakeholder influence over decisions. In the context of MSEs, despite possessing high legitimacy and urgency (dependent stakeholder), athletes typically lack the power necessary to significantly influence organizational outcomes (13, 14). Without the ability to direct organizational action (power), athlete perspectives may be recognized but not enacted. This can leave athlete-centredness as a rhetorical ideal. Despite increasing recognition of athlete-centredness within sport governance, there remains limited understanding of *how* athlete influence is operationalized and enacted within governance structures (15, 16). As such, researchers have called for more robust empirical research to better understand how athlete voices are incorporated into elite sports governance (4).

The Toronto 2015 Pan/Parapan American Games provide a unique case through which to examine the implementation of an athlete-centred approach. As a component in their bid, TO2015 identified the goal of having an “overall athlete-centred approach to the Games” (17). From this, an Athlete Advisory Council (AAC) was established early in the process to officially embed athlete perspectives in the management of the MSE. The aim of this study is to examine how athlete-centred governance was understood, constructed, and enacted through AAC processes over time. This study demonstrates that athlete-centred governance within an MSE occurs through embedded epistemic influence and structured operational integration rather than formal redistribution of decision-making power. Using interpretive process tracing, the study advances a five-phase lifecycle model demonstrating how the influence of dependent stakeholders became increasingly institutionalized while formal authority remained institutionally constrained. In this case, athlete-centred governance is understood as a process of bounded empowerment, by which athlete legitimacy, urgency, and operational integration expanded without corresponding redistribution of formal decision-making authority.

## 2 Methods

A qualitative case study was adopted to examine the implementation of athlete-centred governance within an MSE, using documentary analysis (18–20). Interpretive process tracing (19–22, 43) was used to examine how athlete voice became embedded within the decision-making, governance, and implementation of TO2015. Interpretive process tracing was selected because the study sought to reconstruct how athlete-centred governance was understood, constructed, and enacted in TO2015. Specifically, the study examines (1) the establishment of the AAC; (2) mechanisms of athlete engagement within the organization; and (3) the AAC's outputs. The analysis focuses on processes of legitimization, representation, and translation through which athlete-centredness was incorporated into organizational practice (15, 16, 23). Following interpretive research practices, governance was treated as an ongoing process of meaning-making, organizational translation, and enactment rather than a causal system.

This approach was employed to reconstruct how meanings and governance developed over the course of the AAC life cycle and did not involve causal testing (18–20). The research traces how perspectives of the AAC were communicated, interpreted, and incorporated into organizational decision-making. It places considerable emphasis on achieving contextual consistency and plausibility in interpretation (18–20). As the vice-chair of the AAC, the researcher was concurrently a doctoral candidate and was able to engage in recommendations and aspects of implementation. This position provided privileged insights into governance, including informal governance dynamics and non-documented decision-making, that would otherwise be unavailable through any other means (19). Given the researcher's dual role in the AAC, great emphasis was placed on the effect that proximity to the organizational processes and relations might have on the interpretative assumptions made about influence, governance dynamics and decision-making processes. In cases where independent sources of documentation were limited, the observations were treated as interpretive rather than evidentiary. Reflexive memoing and an audit trail were conducted to capture the analytic decisions and assumptions made throughout the process.

This documentation data set was made up of about 40 documents that were purposefully chosen based on their relevance to the governance process of the AAC. Three hierarchical data levels were established. First was the primary document; this was the AAC white paper (24). This document provided the interpretation and recommendations of the council concerning athlete-centredness, and it is important to note the influence of the document on the process of implementation. Secondly were the secondary documents. These comprised all the operational documents, internal communication, organizational papers, and media that could contextualize and verify the practices related to the AAC's governance processes (25, 26). Thirdly, reflexive insights served as a supplementary source that was considered only when they provided meaningful context to documentary evidence.

An iterative process was applied to the analysis whereby, through repeated engagement with the data, the sequence of key events was reconstructed. Based on this, how athlete-centred meanings were expressed, discussed, and transformed into governance practices was examined. Reflexive memoing was used to identify patterns and recreate key governance processes (19, 20, 27). Interpretive plausibility was strengthened through convergent evidence, audit trail (dependability), and reflexivity (confirmability) (19, 27). In line with interpretive qualitative research, the study prioritized contextual plausibility, reflexive transparency, and analytic coherence over direct reproducibility. Overall, this method offers a transparent and contextual interpretation of how athlete-centred governance was implemented in TO2015 and promotes transferability via analytical generalization (18).

## 3 Results

**Figure 1:**
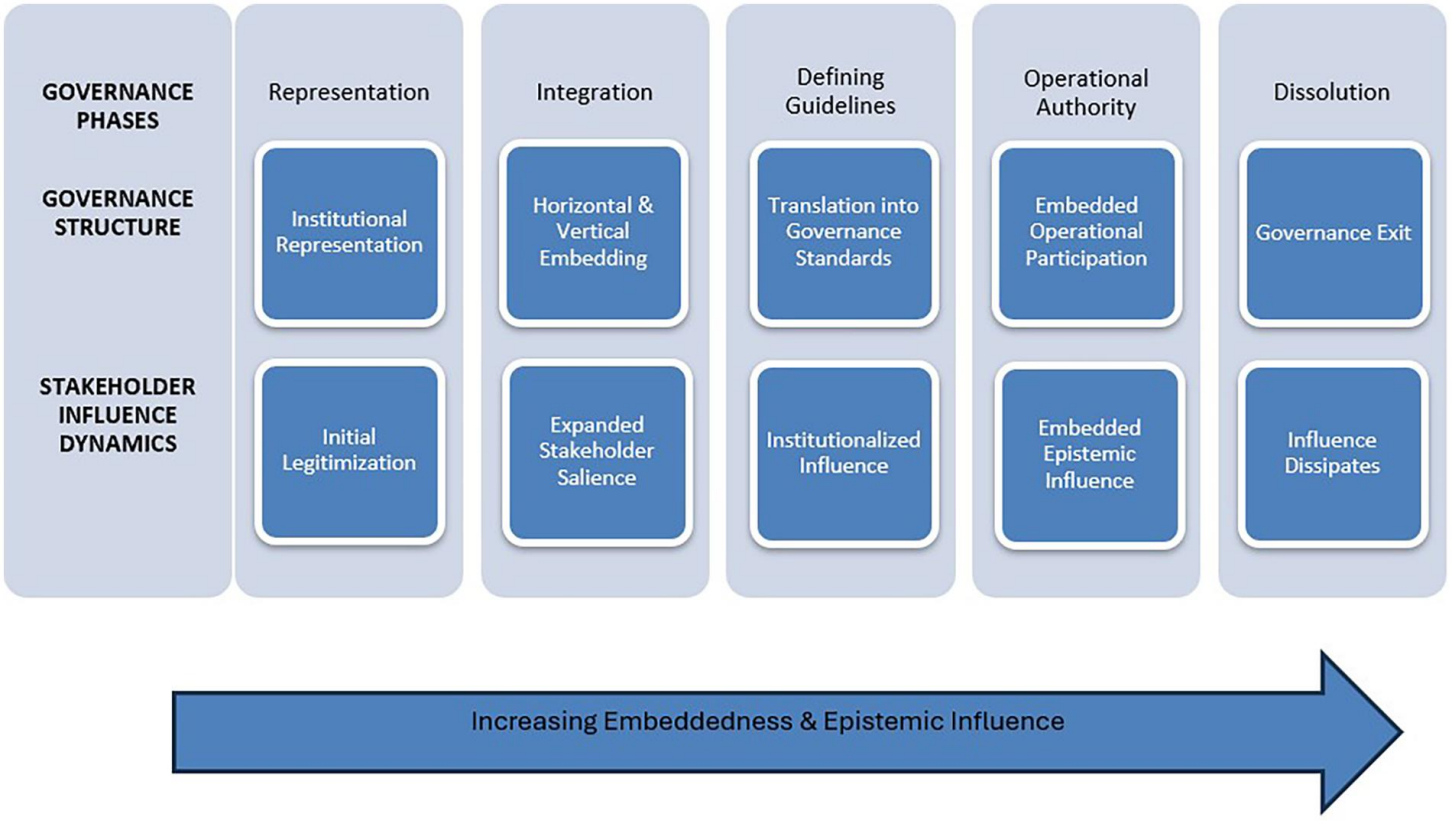
Lifecycle of athlete-centred governance. Note. Formal decision-making authority remained centralized across all phases.

The findings demonstrated a five-phase lifecycle of TO2015's AAC (Figure 1).

### 3.1 Phase 1: representation

The AAC was first established in 2010 within six months of the bid being won by Toronto. It initially consisted of a single para-athlete, who eventually stepped down once the expanded version of the AAC was established. Specifically, in partnership with the Canadian Olympic and Paralympic Committees, nine more athletes were chosen by the board of directors of TO2015 following a competitive national application process to establish initial capacity and representation. Additionally, to ensure a commitment to universal design and representation across diverse sports and professional skillsets, AAC members were purposely selected. The resulting council included five able-bodied athletes and four para-athletes, with over 30 combined appearances at major Games (e.g., Olympic Games and Pan/Parapan American Games). As a group, they had more than 20 medals in major international multi-sport competitions and represented nine unique sport disciplines. AAC members were responsible for communicating the perspective of the TO2015 athletes with the intended goal of enhancing the athlete experience (17, 28). The establishment of the AAC represented the initial institutional legitimization of athlete voice within the governance structure of TO2015. Despite athletes having limited formal authority in this phase, their official representation expanded their stakeholder legitimacy and positioned them to become embedded contributors with time.

### 3.2 Phase 2: integration

The AAC was engaged vertically and horizontally within the TO2015 governance structure. Vertically, the chair, vice-chair, and a para-athlete represented the AAC's views directly to the board of directors. The AAC's influence was not limited to board level reporting; it was also operationalized through an integrated horizontal approach. This was the result of three mechanisms. First, the TO2015 sport director engaged directly with the AAC by maintaining a direct line of communication with the council, attending meetings, and even co-authoring the white paper. This supported a consistent organizational connection. The sport director worked with the council as a technical partner. This allowed for an iterative exchange of information, where AAC recommendations could be confirmed for feasibility, while TO2015 plans could be assessed by the council in real time. This horizontal link facilitated the creation and application of the white paper recommendations addressed within the 2012–2015 planning cycle.

Second, one AAC member held dual roles for TO2015. The council member was also a staff member providing her with the ability to promote the perspectives of the AAC, within her department of marketing and communications (e.g., daily work, staff meetings, and internal communications). Third, each AAC member was assigned to a particular technical area of the Games based on their professional knowledge and interests, such as transportation, medical, and communications. This integration enabled council members to have a better understanding of the TO2015 planning process, while also allowing them to share their viewpoints in the moment. For example, an athlete with a communication background was able to assist the games in the creation of the communication packages for the athletes, before they had been created and issued.

Although TO2015 engaged with the AAC both horizontally and vertically, their recommendations were not always implemented (23, 29). For example, the AAC realized the importance of communication for promoting community buy-in and identified that bad media coverage would be a distraction for the athletes. However, with the election cycle and media scrutiny, TO2015 received more negative publicity leading up to the event. As a result, the marketing and communications department prioritized managing criticism of the Games. In this instance, the AAC's goal of promoting positive public excitement conflicted with the organization's need to focus on risk management. By embedding AAC members into governance and operations, this integration expanded athlete legitimacy and urgency, shifting the council members from being only symbolic in nature to having epistemic influence. However, even as athletes participated in such discussions, institutional power remained centralized.

### 3.3 Phase 3: defining guidelines

One of the first outputs of the AAC was the development of a white paper, “*Defining a Positive TO2015 Athlete Experience*” (24). Reflecting on best and worst experiences at MSEs, and data from the 2010 Commonwealth Games athlete survey, the council identified nine functional areas that directly affect participating athletes (30–32). These included: (1) atmosphere; (2) accommodation; (3) transportation; (4) security; (5) ceremonies; (6) technology; (7) food; (8) communications; and (9) volunteers. For each domain, specific guidelines detailed what a positive experience would entail. For example, the council identified a dedicated “Games Lane” to avoid traffic as being a marker of a positive experience and requested transportation that could accommodate wheelchairs and equipment. Likewise, for accommodation, blackout drapes were identified as a need for athletes to assist with recovery, while overcrowding of athletes in one room was noted as being problematic. These guidelines served as a reference for producing an athlete-centred Games. The presence of AAC members in working groups facilitated the incorporation of the white paper recommendations into decisions such as bed procurement and village catering.

While the white paper offered guidance to fulfilling an athlete-centred Games, it faced some infrastructure constraints. Evidence from late-stage planning meeting notes showed that although the white paper had identified a dining hall too far from residences as being a problem, it was not possible to relocate the planned dining hall location. A remedy to this problem was the introduction of satellite locations, across the village, where athletes could grab food quickly. This suggests the limitations of the white paper through which physical and fiscal legacies restricted the extent to which these recommendations could be implemented (33, 34). The white paper served as a mechanism through which the expertise of athletes was translated into governance guidelines. Athlete-centredness therefore moved from rhetorical commitment toward operational enactment, increasing athlete influence through institutionalized translation processes. Even so, final implementation authority remained constrained by organizational and infrastructural limitations.

### 3.4 Phase 4: operational authority

Once the Games began, the roles of the AAC transitioned to operational leadership as mayors of the Athletes’ Village. In this role, they became a direct conduit to promoting a positive athlete experience on the ground. Mayors of the village engaged in ceremonial activities (e.g., flag raising), encouraged the engagement of athletes, while also addressing any concerns or issues that arose (e.g., visitor guest passes to the Village). As mayors, the AAC was able to conduct daily walkthroughs and assess areas directly affecting athletes such as dining, transportation, and security, while also receiving feedback from the competing athletes. This transition provided the council with direct oversight of the residential and service environments they had helped design. When discrepancies occurred, the council members were able to use their official mayoral standing to interact directly with the organizing committee. Transitioning to the operational leadership roles as mayors of the Athletes’ Village illustrates how the AAC's influence evolved. They shifted from advising on what should be done to becoming part of the process and providing input about what was being done.

### 3.5 Phase 5: dissolution

In the final phase of the AAC's lifecycle, participating athletes’ perceptions of the Games were assessed. The questionnaires served as an audit of the areas identified by the AAC as directly affecting athletes. This provided an evaluative measure of the Games’ success. An executive summary of these results was shared with the organizing committee, prior to the dissolution of the AAC. With the dissolution of the AAC, one of the greatest challenges was the absence of formal mechanisms for knowledge transfer through which informal networks and best practices could be shared with future AACs. The dissolution of the AAC reflects the transient and event-driven nature of athlete-centred governance structures in MSEs. Although the AAC generated knowledge and experience regarding embedded governance, the lack of formal mechanisms for transferring knowledge restricted the longevity of athlete voice in governing beyond the life cycle of the MSE. This dissolution demonstrates how salience and embedded influence remained contingent upon the temporary structure of MSEs. Following the completion of the event, the organizational urgency associated with athlete participation diminished substantially, contributing to the decline of athlete influence within governance processes. These findings suggest that the influence of athletes is transient and not based on an actual shift in power, but on temporary needs of the organization.

## 4 Discussion

TO2015 provides a unique case to examine how athlete-centred governance was understood, constructed, and enacted through AAC processes over time. As illustrated in Figure 1, athlete-centred governance emerged as a structured, multi-level process of inclusion, as athlete stakeholder salience expanded through sustained horizontal and vertical embedding within governance processes. The findings suggest that bounded empowerment operates through conditional forms of stakeholder involvement in which organizations expand athlete voice without formal stakeholder power. Within TO2015, athletes emerged as legitimate contributors to operational planning and governance discussions, particularly when their experiential expertise aligned with organizational objectives and operational constraints. As the AAC became more deeply involved within governance processes, members developed greater coordination and operational familiarity. This enabled the council to function as a coordinated governance actor rather than simply a collection of athlete representatives.

AAC members also developed increasing awareness of the organizational and relational boundaries within which their influence could operate. These parameters were not always formally defined. Instead, they emerged as part of the process of engaging in dialogue with organizational leaders and working within the culture of governance and organization. Consequently, the AAC's influence was mediated through institutional gatekeeping processes that determined when athlete voice was considered actionable or strategically valuable. This reflects a broader governance issue within the stakeholder identification and salience framework, by which organizations may seek the benefits of dependent stakeholder participation while authority for decision-making remains centralized (10–12, 44). Under these conditions, empowerment may not translate into meaningful governance democratization.

Athlete influence extended beyond symbolic representation. Through the incorporation of the AAC in decision-making processes, athlete-centredness was implemented as a governance practice instead of a service delivery mechanism. Vertically, athlete-centredness involved representation through direct reporting to the board through the chair, vice-chair, and para-athlete representation; horizontally, through inclusion in functional units of TO2015. These structures ensured continuous interaction between the AAC and decision makers so that athlete contributions could both influence decision-making and be assessed for feasibility in real time. Empirical evidence from the phases of representation and integration reveals that although athlete voice was legitimized within governance discussions, authority over final decisions remained constrained (23, 29).

Taking into consideration the white paper (17), it is evident that factors affecting athlete performance extend beyond the competitive environment itself. The decision-making process of the organization, such as scheduling programs, transport logistics, and availability of food, may affect either the introduction or prevention of unnecessary physiological and psychological pressure (14, 15). Through the involvement of the AAC, athletes were able to contribute to identifying these environmental pressure points. While athlete voice was integrated across different levels of governance, they lacked formal voting capacity, and final decisions were dependent upon the balancing of other factors that sometimes overrode athlete priorities, even when athlete input materially shaped the range of feasible solutions. These findings also contribute to broader organizational discussions concerning participatory governance, advisory structures, and embedded stakeholder voice within complex institutions.

This study also holds relevance within the contemporary sport governance landscape. Researchers have critiqued how athlete-centred rhetoric, welfare protection, and organizational accountability may be undermined by centralized governance structure, whereby athletes are provided limited influence within the decision-making processes (35, 36). Additionally, sport organizations generally are self-governed, which allows them to set policies, priorities, and apply a top-down approach to governance (35–37). In ‘*The Future of Sport in Canada Commission's Final Report’* (38) the national sport system was characterized as being “broken” and unsustainable, such that athletes recognized their prosumer position, but felt they were treated as “disposable commodities” rather than “valued contributors.” The failure to meaningfully include athlete representation in decision-making was associated with welfare and safeguarding concerns. The TO2015 case provides an empirical example of bounded empowerment, where athlete legitimacy, urgency, and integration of athletes in the operations expanded without an equivalent redistribution of formal organizational authority. Despite the lack of formal voting rights, the integration of the AAC in the governance and operations of TO2015 facilitated the influence of athletes. The findings may therefore help explain why recent Canadian governance reforms have increasingly emphasized not only athlete consultation, but also formalized representation and engagement within governance structures.

Furthermore, it can be argued that bounded empowerment could function as a form of transitional participatory governance, as opposed to being a final stage of athlete-centred governance. Within TO2015, athletes gained increasing legitimacy, operational inclusion expanded, and epistemic influence through their sustained involvement in the organizational processes and planning. However, formal decision-making authority remained centralized across all phases of the AAC's lifecycle. Certainly, formal voting rights are one mechanism through which participation of athletes can become institutionalized into governance structures; but, voting alone may not redistribute power unless athlete representation reaches critical mass to materially influence organizational decision-making (39). Consequently, meaningful governance democratization may depend not only on formal representation, but also on the extent to which governance structures enable collective athlete influence over organizational decision-making in addition to representation within the governance process. Therefore, athlete-centred governance within TO2015 functioned as a contingent form of participatory governance in TO2015. Here, the athlete voice became integrated into governance as a result of sustained involvement, receptiveness, and participation within its operations.

## 5 Policy and practice implications

Based on these findings, and consistent with broader governance research in sport (6, 40), athlete-centred governance can be understood through representation, integration, influence, and constraint, all of which are interrelated. For policymakers and organizing committees of MSEs, the TO2015 model offers several considerations.

1. *Institutionalizing Athlete Representation.* Athlete voice is more likely to be incorporated when it is embedded within formal governance structures, such as commissions or advisory bodies. Providing athletes with formal voting roles may further strengthen their influence.
2. *Selection of Athletes*. Athlete representation may benefit from including a diverse group, with experience across multiple MSEs and professional expertise related to functional areas of the event. This ensures a body of expertise aligned with organizational and operational demands.
3. *Early and Continuous Engagement.* Athlete voice should be incorporated during early planning phases to increase their relevance to operational planning and policy design, rather than relying on late-stage consultation.
4. *Domain-Specific Integration.* The engagement of athletes should extend across all operational areas directly impacting participating athletes to promote targeted contributions and mitigate environmental stressors. Aligning athlete expertise accordingly can strengthen this approach.
5. *Balancing Athlete Integration and Decision-Making Authority.* Future adoption of athlete-centred governance models within the MSE context may be more successful by considering the interaction between integrated advisory participation and the decision-making power. While integrated representation may increase responsiveness and support feasibility testing, some contexts may require more formalized decision-making authority. Forms of influence that operate through informal, relational, or discretionary channels may risk inconsistency, limited transparency, and reduced accountability regarding athlete impact.
6. *Transfer of knowledge.* To ensure knowledge gained by an AAC is not lost, best practices and governance insights should be captured and transferred to future MSEs and AACs.
7. *Process-Oriented Evaluation.* Evaluating how athlete input is incorporated into decision-making processes may provide a more accurate assessment of athlete-centred governance than outcome measures alone.

These recommendations position athlete-centredness as a matter of deliberate governance, rather than simply a reflection of service quality or a post-event satisfaction outcome.

## 6 Limitations and future directions

There are several limitations that should be considered, including generalizability. This study examines a single case; therefore, findings may vary across MSE contexts with different governance structures and organizational constraints (18). Although the lifecycle phases are presented sequentially in this case, future governance systems may experience overlaps or variation depending on institutional context and organizational constraints. Further, this study focuses on the processes of athlete-centred governance and not the participating athletes’ perceptions. Therefore, caution should be applied when interpreting the athlete experience as it relates to this study (41, 42). Finally, the researcher's dual role introduces potential observer bias, particularly with regards to interpreting the extent of the AAC's effect within decision-making processes. Despite the employment of reflexivity and triangulation, this remains an inherent limitation of the study (19).

It is recommended that future research expand on this study by comparing governance systems across multiple MSEs (12, 39). Researchers should examine how different configurations of athlete inclusion produce varying forms of influence – ranging from formal authority to embedded and epistemic forms of power. Additionally, longitudinal studies should examine how athlete-centred governance structures evolve beyond the lifecycle of an MSE, accounting for transfer of knowledge and institutional learning (14, 23).

## Data Availability

The data analyzed in this study is subject to the following licenses/restrictions: The data sets presented in this article are not readily available due to confidentiality and/or ethical restrictions. Requests to access the datasets should be directed to the corresponding author. Requests to access these datasets should be directed to Nicole Forrester, nforrester@torontomu.ca.
